# Combining Music and Indoor Spatial Factors Helps to Improve College Students’ Emotion During Communication

**DOI:** 10.3389/fpsyg.2021.703908

**Published:** 2021-09-14

**Authors:** Jiani Jiang, Qi Meng, Jingtao Ji

**Affiliations:** Key Laboratory of Cold Region Urban and Rural Human Settlement Environment Science and Technology, Ministry of Industry and Information Technology, School of Architecture, Harbin Institute of Technology, Harbin, China

**Keywords:** social interaction, college students, audiovisual interaction, music, emotion during communication, pleasure-arousal-dominance emotional state model, spatial factors, social characteristics

## Abstract

Against the background of weakening face-to-face social interaction, the mental health of college students deserves attention. There are few existing studies on the impact of audiovisual interaction on interactive behavior, especially emotional perception in specific spaces. This study aims to indicate whether the perception of one’s music environment has influence on college students’ emotion during communication in different indoor conditions including spatial function, visual and sound atmospheres, and interior furnishings. The three-dimensional pleasure–arousal–dominance (PAD) emotional model was used to evaluate the changes of emotions before and after communication. An acoustic environmental measurement was performed and the evaluations of emotion during communication was investigated by a questionnaire survey with 331 participants at six experimental sites [including a classroom (CR), a learning corridor (LC), a coffee shop (CS), a fast food restaurant (FFR), a dormitory (DT), and a living room(LR)], the following results were found: Firstly, the results in different functional spaces showed no significant effect of music on communication or emotional states during communication. Secondly, the average score of the musical evaluation was 1.09 higher in the warm-toned space compared to the cold-toned space. Thirdly, the differences in the effects of music on emotion during communication in different sound environments were significant and pleasure, arousal, and dominance could be efficiently enhanced by music in the quiet space. Fourthly, dominance was 0.63 higher in the minimally furnished space. Finally, we also investigated influence of social characteristics on the effect of music on communication in different indoor spaces, in terms of the intimacy level, the gender combination, and the group size. For instance, when there are more than two communicators in the dining space, pleasure and arousal can be efficiently enhanced by music. This study shows that combining the sound environment with spatial factors (for example, the visual and sound atmosphere) and the interior furnishings can be an effective design strategy for promoting social interaction in indoor spaces.

## Introduction

There has been a weakening of social interaction among contemporary college students, many of whom prefer chatting online as opposed to face-to-face communication. According to The 47th China statistical report on Internet Development (2021), by December 2020, netizens aged 20–29 accounted for 17.8% of total users, and students accounted for the highest proportion in the occupational structure. College is a period characterized by dynamic development of the brain and strong interactions within the social environment. Students are vulnerable to a range of psychological problems; three-quarters of cases of four common mental disorders begin between the ages of 20 and 30 (Patel et al., 2007; Mirón et al., 2019). Research has shown that social anxiety is a significant and common issue for college students, given the growing range of academic and social stressors (Evans et al., 2018; Yang et al., 2019). Addiction to online networks can impair social activities, work/study, interpersonal relationships, and/or psychological health and well-being (Andreassen and Pallesen, 2014), and significant amounts of screen time correlate with both depression and anxiety (Lin et al., 2016; Rosenthal et al., 2021). As good interpersonal communication ability is one of the most important qualities of college students, the use of architectural design to improve communication spaces and promote interaction is of great significance. However, architectural methods of promoting communicative behavior have so far remained at the level of spatial design and have neglected the architectural sound environment.

Human actions, particularly in face-to-face encounters, invite responsive behaviors that include variations in wording, stress, volume, tone of voice, gesture, gaze, head movements, and even breathing patterns (Jensen and Pedersen, 2016). The social interaction model indicates that senders’ displays of emotion provide powerful signals to receivers during interpersonal interactions, which people are hard-wired to pick up on and then rely on to guide their own behavior (Côté, 2005). Furthermore, interpersonal relationships can be fulfilled by emotions (Wubben et al., 2009). Authentic displays of some emotions – for instance, happiness – communicate an intention to affiliate and indicate that the individual is friendly and agreeable (Côté, 2005). The concept of emotional contagion (Hatfield et al., 1993) entails a ripple effect (also known as an imitation effect) of human interaction through conscious or unconscious induction of emotional states and behavioral attitudes (Hess and Blairy, 2001). For instance, negative emotions, especially anger, can often lead to violence (Umberson et al., 2003); conversely, regarding positive emotions, positive correlations have been found between the total amount of face-to-face interaction and the interlocutors’ resulting mood (Ono et al., 2012). Communication is a fundamental part of social face-to-face interaction that can produce cooperation or coordination (Olguin et al., 2009). However, there is insufficient research focusing on how to balance emotions during communication in combination with the sound environment.

Previous studies have shown that emotion can be affected by environmental contexts (Li et al., 2012; Lazarus, 2014), including luminous, thermal, and acoustic factors. In terms of acoustic perception, the soundscape is defined as a sonic environment, with an emphasis on the way it is perceived and understood by individuals or by society (Brown et al., 2011). Evaluations of stimuli indicate that human emotions track changes in environmental sounds, especially in speech and music (Ma and Thompson, 2016). As a specific sound source, music has been found to be beneficial for rewards, motivation, pleasure, stress, arousal, immunity, and social affiliations (Juslin and Västfjäll, 2008; Chanda and Levitin, 2013). Evidence against a strict cognitivist position suggests that music can induce some sort of emotional response (Hunter and Schellenberg, 2010), which can be categorized as one of the nine common emotions of wonder, transcendence, tenderness, sadness, nostalgia, peacefulness, power, joy, and tension (Huron, 2011). Previous studies have found a modulation of the activities of the brain’s core structures ascribed to emotion processing by music, involving the amygdala and the hippocampus, which are central elements in the network that process emotions such as happiness, anxiety, anger, and annoyance, as well as for assessing facial expressions and thereby contributing to communication, social behavior, and memory (Koelsch and Skouras, 2014; Hans-Eckhardt, 2017). For instance, music can lead to emotional contagion; happy music triggers the zygomatic muscle for smiling, together with an increase in skin conductance and breathing rate, whereas sad music activates the corrugator muscle (Koelsch, 2014). Despite these valuable findings, most research on music-evoked emotion has been conducted under laboratory conditions without taking into account the broader context, including spatial conditions and the physical environment. In addition, most studies have focused on the individual emotional state to the neglect of social interactions in specific patterns of behavior.

Being physically, emotionally, and psychologically aware of the space, we occupy is a feeling that can be described as being present. Jürgen Joedicke noted the need to take into consideration, the experience of space as well as spatial perception (Vasilski, 2016). Indoor space is the main site for human activities. Interaction between people and their located environment, like social interaction, is spontaneous and unavoidable, and the specific psychological emotions evoked by the physical and environmental attributes of personal interior space offer a highly interesting topic for research (Reddy et al., 2012). Interrelated elements of interior design, including spatial form, structure, light, texture, and color, as well as environmental factors such as lighting, sound, temperature, and humidity, affect spatial atmosphere and emotion (Reddy et al., 2012).

Taking spatial form as an example, a study of the impact of office design on absence rates has shown that stress levels and sick leave rates are higher in traditional open-plan offices than in cell-offices or combi-offices (Danielsson et al., 2014). Numerous studies have also examined the effects of light on emotion. For instance, the use in accent lighting of saturated blue and cyan colors with a color temperature of 5,000–5,500K has been found to lead to the emotion of liveliness (Kim and Mansfield, 2021). In terms of color, red is likely to be stimulating because it increases blood pressure and heart rate (Manav, 2017). Natural communication is dynamic in nature, hence the importance of investigating the audiovisual effects of space on social interaction (Boer et al., 2018). However, most studies of emotional perception have relied on the visual factors of spaces and ignored the combined sound environment.

Previous work in a classroom context analyzing the effects of music on conversational interaction has shown that, to a certain degree, musical sound has a masking effect on other noises and promotes communication in general (Jiang et al., 2019). Here, emotion during communication refers to changes in emotion following communication compared to the original emotional state before communication. The study utilizes the three-dimensional pleasure–arousal–dominance (PAD) emotional model to assess the emotions of participants through three dimensions of pleasure, arousal, and dominance before and after communication. The difference values (*d*-values) of pleasure, arousal, and dominance are used to reflect the changes in emotion during communication. To investigate differences in the effects of music on emotion during communication at different sites, this study sets out four hypotheses.

Hypothesis 1: is that the effects of music on emotion during communication vary in spaces with different functions.

Hypothesis 2: is that visual atmosphere influences the effects of music on emotion during communication.

Hypothesis 3: is that the sound atmosphere influences the effects of music on emotion during communication.

Hypothesis 4: is that interior furnishings serve as an influential spatial factor that moderate the effects of music on communication.

Hypothesis 5: is that the effects of music on emotion during communication differ when participants are subject to different social characteristics, including the intimacy level, gender combination, and group size.

## Methodology

### Experimental Site

**Figure 1:**
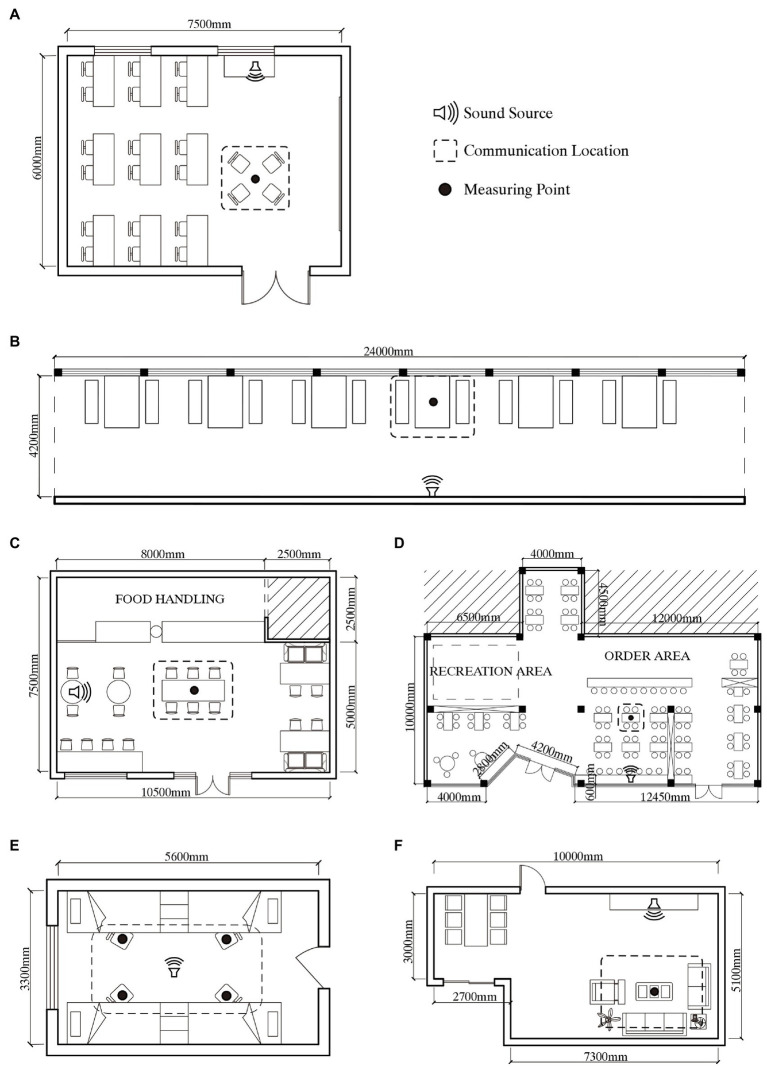
Indoor layouts of the experimental sites: **(A)** CR, classroom; **(B)** LC, learning corridor; **(C)** CS, coffee shop; **(D)** FFR, fast food restaurant; **(E)** DT, dormitory; and **(F)** LR, living room.

There are two main kinds of emotion-related experiments: field experiments and laboratory experiments. On the basis of reliability and authenticity (Harrison, 2003), in this study, a field experiment was conducted in different sites, respectively, under the same musical environment. In terms of the selection of experimental sites, the location and time to confront people (especially strangers) can significantly influence the communication that takes place (Weisburd, 2021). In order to avoid any influence of familiarity with the space on communication (Krupa et al., 2018), indoor places frequently visited by students were chosen for this study. In line with common student activities, experimental sites were classified into learning, dining, and residential spaces based on spatial functions (Boukhechba et al., 2018). In order to explore the influence of qualities-related factors on emotion (Maheshwari et al., 2019), such as visual and sound atmospheres, as well as interior furnishings, six typical indoor communication spaces were chosen as the experimental sites and were compared one by one based on their functional classification: classroom (CR), learning corridor (LC), coffee shop (CS), fast food restaurant (FFR), dormitory (DT), and living room (LR). Indoor layouts of the experimental sites are shown in Figures 1A–F. LC here refers to a corridor containing a learning space as shown in Figure 1. Tables and chairs arranged in the learning space are used for students’ daily reading and communication. And, it is worth mentioning that the learning space (CR, LC) discussed in this study is used for students’ self-study (not teaching), including learning communication and chat.

In terms of behavior mode, the CR and LC are used for self-study, and typical behaviors in these areas include learning communication and daily chat; the CS is frequently visited by students for socializing and tea breaks; FFR is a popular location for dining; and the DT (four-person) and LR in residence halls comprise the main living areas for college students, wherein typical behaviors include rest, leisure, and socializing.

A thermal measurement instrument and illuminance meter were used to ensure that indoor temperature and illumination were within the comfort range, and therefore, not likely to influence emotion or performance (Altomonte, 2017; Petersen and Knudsen, 2017). In terms of thermal factors, related experimental results have shown that performance improves as the indoor temperature approaches 23°C (Nematchoua et al., 2019) and decreases when the temperature rises above 25°C (Niemelä et al., 2002). Therefore, the experiment time was set from 9a.m. to 4p.m. to maintain the indoor temperature at 23–25°C. In terms of luminous factors, previous studies have shown that illuminance and light color can affect fatigue and emotion (Carlucci et al., 2015). Preferred illuminance was therefore set in the range of 310–600lux, and a neutral white (4,000K) space was used (Bowers et al., 2010; Hidayetoglu et al., 2012; Yang and Moon, 2018). Since it was difficult to maintain the same illuminance on all six sites, neutral white (4,000K) light was used to decrease the effects of the light environment, and lamps in the room maintained an average illuminance of 310–600lux.

Sound level meters (BSWA801, BSWA, Beijing, China) were located at the measurement points shown in Figure 1 and at a height of 1.2m from the ground, and provided multiple measures of the background sound pressure level (SPL) at each of the six experimental sites for 5min per hour from 9a.m. to 4p.m., with recordings every 5s, which were A-weighted in the uncontrolled condition without subjects (Torija et al., 2012). In addition, because the experimental behavior was face-to-face communication, reverberation time (RT) was a significant factor influencing speech intelligibility (Yang and Hodgson, 2006), which may influence the quality of communication. Therefore, using the Eyring formula (Passero and Zannin, 2010), the RT of each experimental site was calculated, including the absorption coefficient, area, and amount.

The characteristics of the experimental sites, including scale, background SPL, RT, and behavior patterns, are shown in Table 1. The data show that the average A-weighted equivalent SPL for 5min of background sound recording at the measurement point ranged from 32.5 to 63.2dBA at the six sites, among which, under the same spatial function, the difference in SPL was largest (25.3dBA) between the FFR and the CS. The sensitivity of the instrument was ±0.5dBA. In addition, the average RT value at each site with 2–5 participants was provided.

**Table 1:** Basic spatial and acoustic information of six experimental sites.

Experimental site	Indoor area (m^2^)	Floor height (m)	Color scheme	Background SPL (dBA)	RT (s)	Behavior mode
CR	45	3.5	White, Wooden, and Gray	32.5	1.16	Learning, chatting
LC	100	3.0	White, Dark, and Gray	36.7	1.54	Learning, chatting, and walking
CS	72	3.0	White, Wooden, and Yellow	37.9	1.22	Dining, chatting, and dating
FFR	235	3.5	White, Wooden, and Red	63.2	1.41	Dining, chatting
DT	18	3.3	White, Wooden, and Blue	33.9	0.71	Sleeping, learning, and chatting
LR	45	2.8	White, Wooden, and Gray	35.6	0.91	Resting, chatting

### Experimental Music

Previous research indicates that tempo, SPL, and musical emotions may also influence emotion (Hunter and Schellenberg, 2010); this phenomenon that has been assessed using behavioral, physiological, and neurological measures (Koneni, 2008). In terms of tempo, fast music can increase brain activity (Nicolaou et al., 2017). To avoid interference from the high arousal effect of music on the differences between spatial types, slow music was used in this study. Music with high SPL can also increase perceived activation and tension (Olsen et al., 2015). Excluding the FFR, the background SPLs of the other five sites were all below 40dBA; in order to ensure the masking effect of music as well as the sound comfort (Jiang et al., 2019; Meng et al., 2020), an SPL of 50dBA was adopted for the present study.

Musical emotional characteristics can be classified in terms of musical dimensions, such as pitch height, loudness, timbre, tempo, and intensity (Goshvarpour et al., 2017), and grouped into synonym clusters, such as happiness, sadness, fear, and neutral (Paquette et al., 2018). Related studies have shown that peacefulness is a complex emotion that overlaps with each quadrant of a circumplex model defined by the dimensions of arousal and valence (Hunter and Schellenberg, 2010) without clear emotional directivity. Thus, taking into account the evaluation of familiarity and liking by the college students (Meng et al., 2020), *A Comme Amour* (slow tempo, peaceful, 50dBA, high degree of familiarity and liking) was chosen on the basis of restricted gene expression programming (Zhang and Sun, 2013) as the experimental music in this study. As the CR had the lowest background SPL of the six sites, the SPL of the experimental music was measured there. Under the condition with music, measurements were taken three times for 5min each time, at 1-min intervals, with readings every 1s, which were A-weighted (Crandell et al., 2004). Readings for the three times were averaged and recorded. To ensure the accuracy of the musical SPL, the volume of music was adjusted until the average reading of musical SPL reached 50dBA (actual data 49.66±0.5dBA, denoted in what follows as 50dBA).

### Participants

In order to avoid the effect of identities and expectations on participants’ satisfaction with communication, all participants were graduate students or undergraduate students of the university (Gudykunst and Shapiro, 1996). Further, to avoid potential confounding effects of formal training in music (Gabrielsson, 2002), all participants were non-music majors and had never undergone formal training in music. Before starting the experiment, participants were asked to maintain their emotional stability, obtain sufficient sleep, and follow the same routine as they would during the survey period, without any interference from other events such as exams or parties. To ensure adequate statistical power, G*Power (a general power analysis program) was used to analyze the minimum sample size of subjects, assuming an effect size of *d*=0.5, *α*=0.05 and Power (1−β)=0.8. To answer the main research question regarding the differences between different indoor spaces, the minimum average required sample size was 51 for the student’s *t*-test (between-group) and 53 for one-way ANOVA (between-group) when there were three groups. Information about the participants is given in Table 2. Of students in the sample, 55 participated in the CR, 55 in the LC, 52 in the CS, 59 in the FFR, 54 in the DT, and 56 in the LR.

**Table 2:** Basic information on participants by experimental site.

Experimental site	Total sample size	Gender	Sample size	Mean age	SD of age
CR	55	Male	27	23.96	1.48
		Female	28	23.75	1.71
LC	55	Male	26	23.62	2.74
		Female	29	23.14	2.46
CS	52	Male	22	24.45	1.99
		Female	30	24.20	1.95
FFR	59	Male	24	24.54	3.79
		Female	35	24.14	2.56
DT	54	Male	27	24.48	1.83
		Female	27	24.70	0.95
LR	56	Male	25	24.56	1.53
		Female	31	24.48	1.41

### Emotional Model

Recognition and analysis of human emotions have been researched extensively in neuroscience, psychology, cognitive science, and computer science. Mainstream research on human emotion has focused on facial and vocal expressions (Gunes and Pantic, 2010). In terms of the models of perceived affective quality on soundscapes, Ma et al. (2018) illustrated the feasibility of the semantic differential method (SDM) for measuring human perceptions of sound. The quantitative measurements of the subjective meaning of things were obtained from the subjects’ ratings on the bipolar adjective pairs (APs) formed by descriptors with two opposite meanings (Ma et al., 2018). The APs provided a general picture of human perceptions of the tested objects and facilitated comparison between the objects (Ma et al., 2018). Besides, Torresin et al. (2020) indicated that many of perceptional dimensions in complex acoustic environments (i.e., multiple sound types) could be coherently explained under Russel’s circumplex model of affect. In order to indicate the audiovisual effects of music and spatial factors on emotion, an emotional model based on the SDM evaluation system describing emotional states was chosen in this study. The discrete emotional model and the dimensional emotion model are the most widely used models that can effectively express and quantify emotion. The discrete emotion model applies emotional labels (e.g., happiness, sadness, surprise, fear, anger, and disgust), although, a single label may not reflect the complexity of the affective state conveyed by such rich sources of information (Gunes and Pantic, 2010). Instead of applying discrete categories of emotion, the dimensional emotional model analyzes and interprets the subtlety, complexity, and continuity of affective behavior in terms of latent dimensions (Havlena et al., 2010). Compared to a two-dimensional scheme – for example, arousal-valence space model of Thayer and Mcnally (1992) – the three-dimensional solution is more informative and helps to differentiate between what the cluster analysis suggests are separate basic-emotion categories (Shaver et al., 1987). Therefore, three-dimensional PAD emotional model of Mehrabian and Russell (1974) was selected for use in this study. The PAD emotional model consists of three APs, including pleasure–displeasure (P), arousal–nonarousal (A), and dominance–submissiveness (D), among which P is defined as positive vs. negative affective states, with higher evaluations of stimuli being associated with greater pleasure induced by the stimuli; A equates to judgments of high–low stimulus activity, which is defined in terms of the level of mental alertness and physical activity; and D is defined as a feeling of control and influence over one’s surroundings and others vs. feeling controlled or influenced by situations and others (Mehrabian, 1996; Zhang et al., 2007). Gärling et al. (2020) proposed that self-reports on momentary states (e.g., “How do you feel right now?”) in the context of emotional well-being are valid and reliable. In the current study, considering differences in the emotional states of the participants before the experiment, the PAD three-dimensional evaluation was conducted before and after the communication, respectively, and *d*-values of pleasure, arousal, and dominance were used to reflect changes in emotion during communication.

### Questionnaire Design

A questionnaire was used to evaluate the participants’ emotional state with a high level of reliability, because self-reports have been found to be an appropriate and natural method for studying emotional responses to music (Gabrielsson, 2002). The questionnaire consisted of four parts: basic information, overall music evaluation, overall spatial evaluation, and emotion during communication before and after the experiment (based on the three-dimensional PAD emotional model). The structure and descriptions of questionnaire are shown in Table 3; among them, overall musical evaluation represents the effect of music on communication and indicates participants’ preference regarding the presence of music at different sites during communication. Overall spatial evaluation is composed of four spatial factors: spatial scale, perceived spatial color, sound atmosphere, and interior furnishings. Emotion during communication is evaluated from three dimensions: pleasure, arousal, and dominance. The *d*-values of pleasure, arousal, and dominance before and after the experiments are used to reflect the change in emotion during communication.

**Table 3:** The structure and descriptions of questionnaire.

The structure of questionnaire	Question	Description	
Basic information	Name, gender, age, intimacy level and group size.	Intimacy level (1 to 3): stranger (1), acquaintance (2), close friend (3)Group size (1/2): one-on-one (1), multi participants (2)	
Overall musical evaluation	How do you think the music influenced the communication?	No effect (0): Music has no effect. Stimulating (1–10): Music is a little – very stimulating to communication. Distracting (−1 to −10): Music is a little – very distracting to communication.Distracting (−1 to −10): Music is a little— very distracting to communication.	
	(21 points scale)		
Overall spatial evaluation	Please evaluate the space you are located from four aspects.	Spatial scale (1/2): small scale (1), large scale(2)	
		Perceived spatial color (1/2): cold-toned (1), warm-toned (2)	
	(either-or questions)		
		Sound atmosphere (1/2): quiet (1), noisy (2)	
		Interior furnishings (1/2): minimal (1), abundant (2)	
Emotion during communication	Please describe your current emotional state from three aspects (P, A, and D)	Pleasure (1–10):	Depressed to satisfied; Unhappy to happy;
			Restless to comfortable; Angry to content
		Arousal (1–10):	Peaceful to fevered; Unexcited to excited;
	(10 points scale)		
			Relaxed to stimulated; Drowsy to alert
		Dominance (1–10):	Passive to active; Controlled to uncontrolled

### Experimental Design

In order to explore the effects of different spatial types on emotion during communication against a background of music, experiments were conducted in six typical indoor spaces. In terms of experimental means, random assignment, which has high internal validity (Morgan et al., 2000), was used to eliminate systematic differences between the treatment and control groups. Participants recruited on campus were allocated at random to groups of 2–5 people in each conversation. In addition, three levels of communicator intimacy were delineated, including stranger (all sites except DR and LR), acquaintance (all sites), and close friend (all sites). Because the background SPL was different at each of the six sites, to ensure a consistent musical environment, *A Comme Amour* (50dBA) as measured in the classroom was played at the same volume at each of the six sites to ensure the sound pressure of the music was same at each indoor space. In terms of the duration of communication, previous studies have found 5min to be appropriate for an analysis of communication using average and minimum chatting times (Yamaguchi et al., 2012). To avoid duration affecting people’s focused attention and emotion during communication (Dong and Wyer, 2014), each communication period was limited to 5min. The need for participants to communicate with strangers for 5min was mentioned when recruiting the volunteers, so that we could ensure all of the participants showed a strong desire to conduct conversations with others, including strangers. In addition, participants’ post-experiment feedback showed that interactions felt natural and fluent rather than artificial.

**Figure 2:**
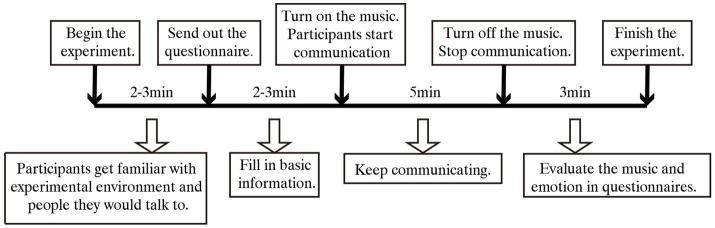
Experimental procedure.

The flow chart of the experiment is shown in Figure 2. Following the random allocation, participants entered the experiment room and took around 2–3min to get familiar with the experimental environment and the people they would talk to. Then, questionnaires were distributed, and participants were required to fill in their basic information according to the instructions in 2–3min. Participants then began to communicate in the music environment, and continued chatting for 5min until the music stopped. During the communication session, considering that participants were strangers, in order to avoid embarrassment several topics were provided for participants to discuss. Participants selected the conversation topics themselves and were then required to complete the questionnaires within 3min, after which the experiment was deemed complete. In addition, some studies have indicated that food may facilitate communication (Dabbs and Janis, 1965; Blouin et al., 2013); therefore, snacks and nonalcoholic beverages were provided in this study.

Regarding the between-subjects design, in terms of spatial function CR, CS, and LR were chosen for comparison. Because the difference in indoor area of these three sites was within 27m^2^, the background SPLs were all below 40dBA, and the colors of the spaces were all warm-toned, the effect of spatial scale and visual and sound environment was deemed minimal. In terms of visual atmosphere, the colors of the LC were cold-toned, which was different from the other spaces. Thus, in order to ensure consistency in the spatial function, CR and LC were chosen for comparison. In terms of sound atmosphere, excluding FFR, the background SPLs of the other five sites were all below 40dBA; thus, in order explore the effect of sound atmosphere of the same functional spaces on communication, CS was chosen for comparison with FFR.

### Statistical Methods

IBM SPSS statistics for Windows, version 23.0 (IBM Corp., Armonk, NY, United States) was used to analyze the relationship between the effects of music on communication and specific spatial factors. The Kolmogorov–Smirnov test was used to analyze the normality of the experimental data – that is, to confirm that the population followed a normal distribution hypothesis. Levene’s test was used for equality of variance, and all variance was found to be equal. According to the number of treatments, ANOVA was used to test for significant differences in spaces with different functions (between-group). Independent *t*-tests were performed to assess differences between spaces with different visual and sound atmospheres and interior furnishings.

## Results

Spatial characteristics are expected to affect the use and feeling of a space, and even the emotions experienced during communication therein. This section presents the results of the analysis of the relationships between the spatial factors and the musical evaluations, average evaluation scores and *d*-values for PAD emotional evaluations in terms of spatial function, visual atmosphere, sound atmosphere, and interior furnishings.

### Spatial Function

**Figure 3:**
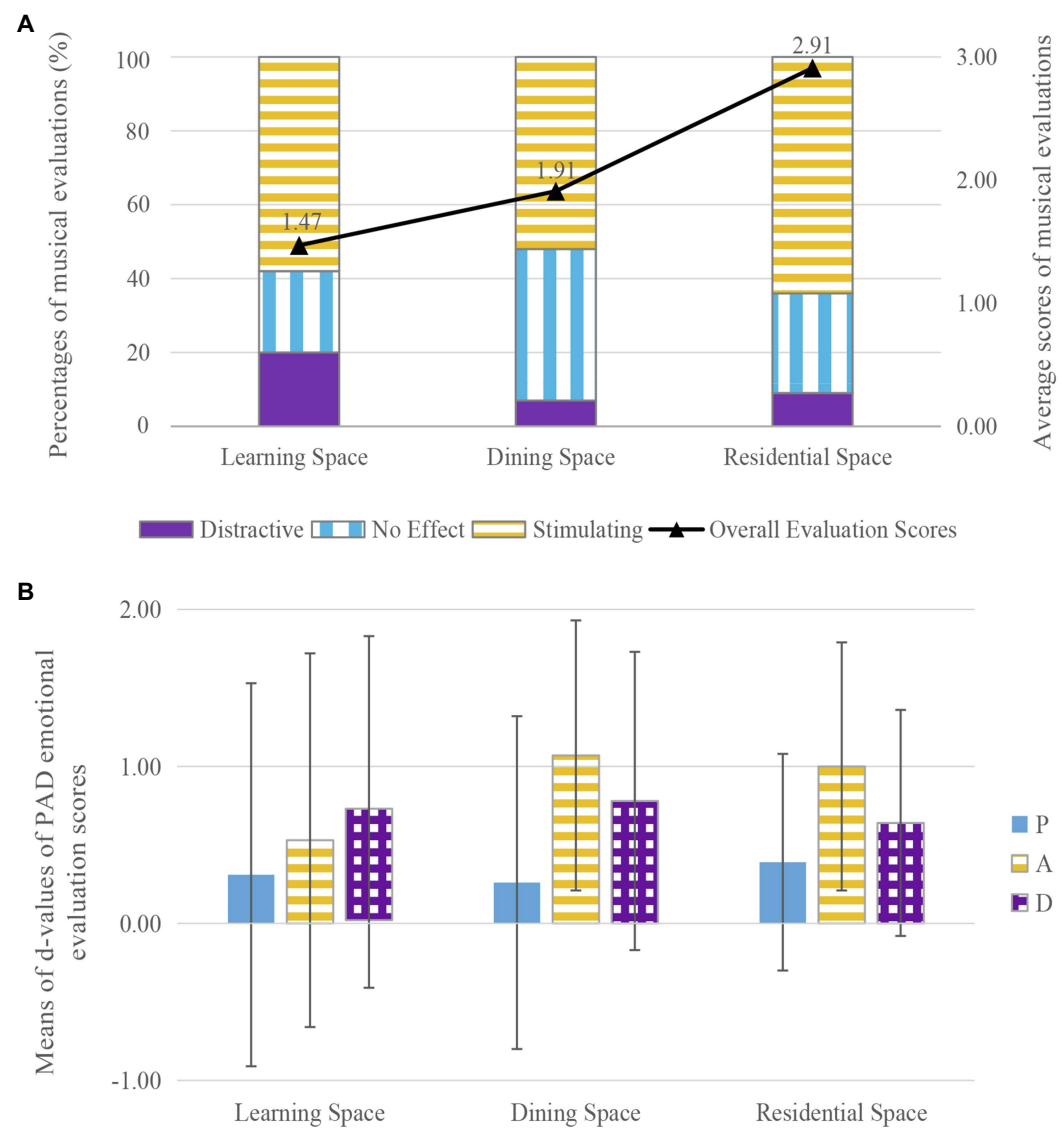
The effects of music on communication in spaces with different functions: **(A)** the effects of music on musical evaluation, **(B)** the effects of music on pleasure–arousal–dominance (PAD) emotional evaluation.

The experimental sites were sorted into three groups according to function (learning spaces, dining spaces, and residential spaces). CR, CS, and LR (all of which were quiet and had a similar indoor area) were chosen for comparison. The relationships between spatial functions and musical evaluations (no effect/stimulating/distracting) and the average scores for musical evaluations are shown in Figure 3A. The percentage who evaluated the music as stimulating to communication ranged from 52 to 64% and was highest in the residential space. The percentage evaluating the music as distracting ranged from 7 to 20% and was highest in the learning space. Average scores for musical evaluation were highest in the residential space (2.91), followed by the dining space, and then the learning space (1.47). Based on the three kinds of spatial functions, the results of the ANOVA show that there was no significant difference in musical evaluation among spaces of different functions (*df*1=2, *df*2=162, *F*=2.225, *p*=0.111, *d*=0.26).

The average *d*-values for the PAD emotional evaluations in spaces of different functions are shown in Figure 3B. Pleasure, arousal, and dominance increased to varying degrees, and the *d*-values of pleasure and dominance were similar across the three spaces. Pleasure increased from 0.26 to 0.39 and was highest in the residential space, whereas dominance increased from 0.64 to 0.78 and was highest in the dining space. The *d*-value for arousal was lowest in the learning space (0.53). The results of the ANOVA show that there were no significant effects of different spatial functions on pleasure (*df*1=2, *df*2=162, *F*=0.061, *p*=0.941, *d*=0.05), arousal (*df*1=2, *df*2=162, *F*=1.309, *p*=0.273, *d*=0.27), or dominance (*df*1=2, *df*2=162, *F*=0.070, *p*=0.933, *d*=0.05).

### Visual Atmosphere

**Figure 4:**
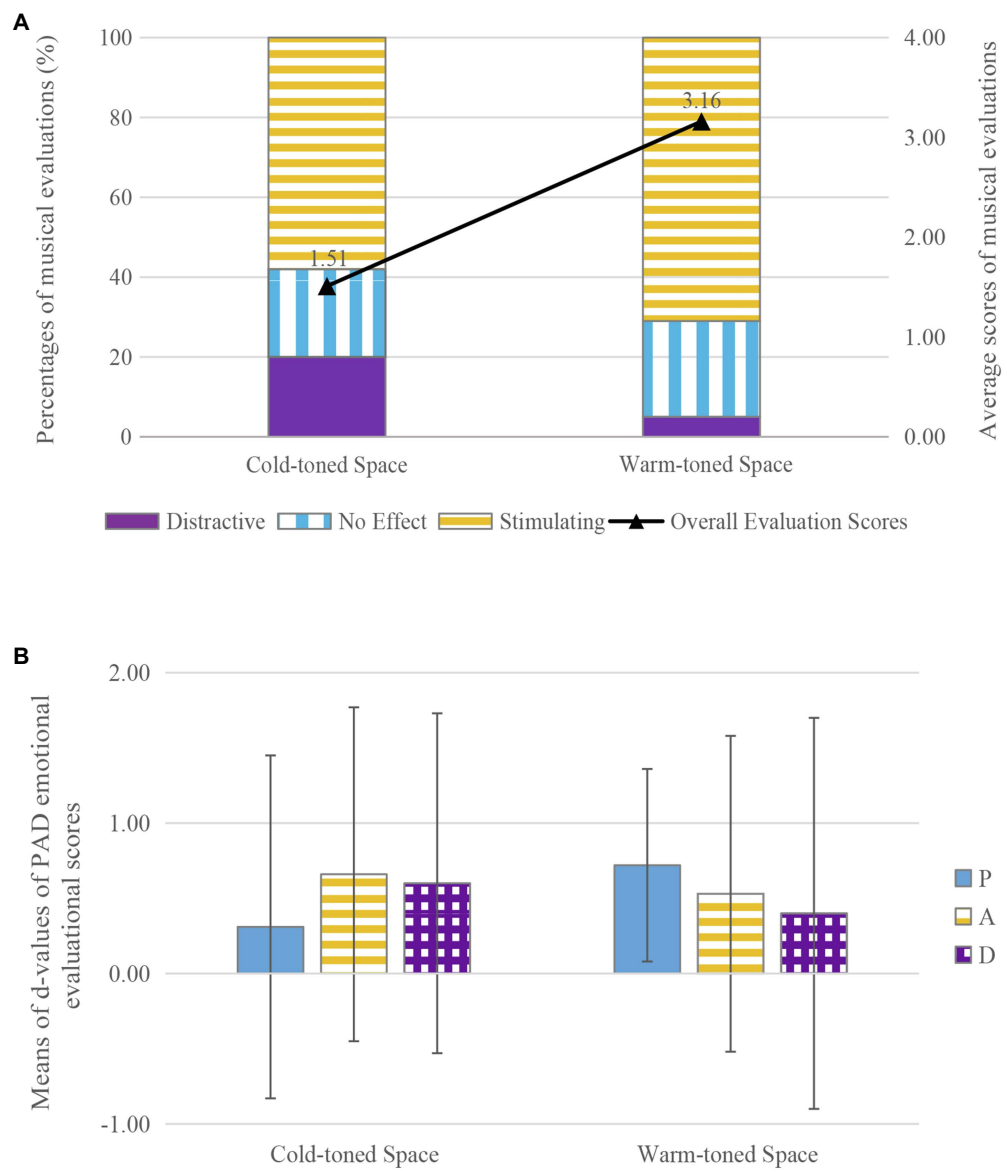
The effects of music on communication in spaces with different visual atmospheres: **(A)** the effects of music on musical evaluation, **(B)** the effects of music on PAD emotional evaluation

In accordance with subjective evaluations of perceived space color, the experimental sites were grouped into cold-toned spaces and warm-toned spaces. CR and LC (which were both quiet and of learning function but had a clear difference in color evaluation) were chosen for comparison. The relationships between visual atmosphere and percentages for the musical evaluations (no effect/stimulating/distracting) and the average scores for the musical evaluations are shown in Figure 4A. The percentage who evaluated the music as stimulating was higher in the warm-toned space (71%) than in the cold-toned space (58%), and the converse applied to the proportion who evaluated the music as distracting. Average scores for musical evaluation were much higher in the warm-toned space (*M*=3.16, *SD*=2.214) than in the cold-toned space (*M*=1.51, *SD*=3.894). The results of the *t*-test showed that there was a significant effect of visual atmosphere on musical evaluation (*t*=−2.534, *df*=108, *p*<0.05, *d*=0.50).

The average *d-*values for PAD emotional evaluations in spaces with different visual atmospheres are shown in Figure 4B. Pleasure, arousal, and dominance all increased to varying degrees. The *d*-values for arousal and dominance were 0.13–0.20 higher in the cold-toned space than in the warm-toned space, while the *d*-value of pleasure in the warm-toned space (*M*=0.72, *SD*=1.278) was higher than in the cold-toned space (*M*=0.31, *SD*=2.284). The results of the *t-*test showed that visual atmosphere did not have a significant effect on pleasure (*df*=108, *t*=−1.066, *p*=0.289, *d*=0.21), arousal (*df*=108, *t*=0.285, *p*=0.776, *d*=0.06), or dominance (*df*=108, *t*=0.508, *p*=0.613, *d*=0.10).

### Sound Atmosphere

**Figure 5:**
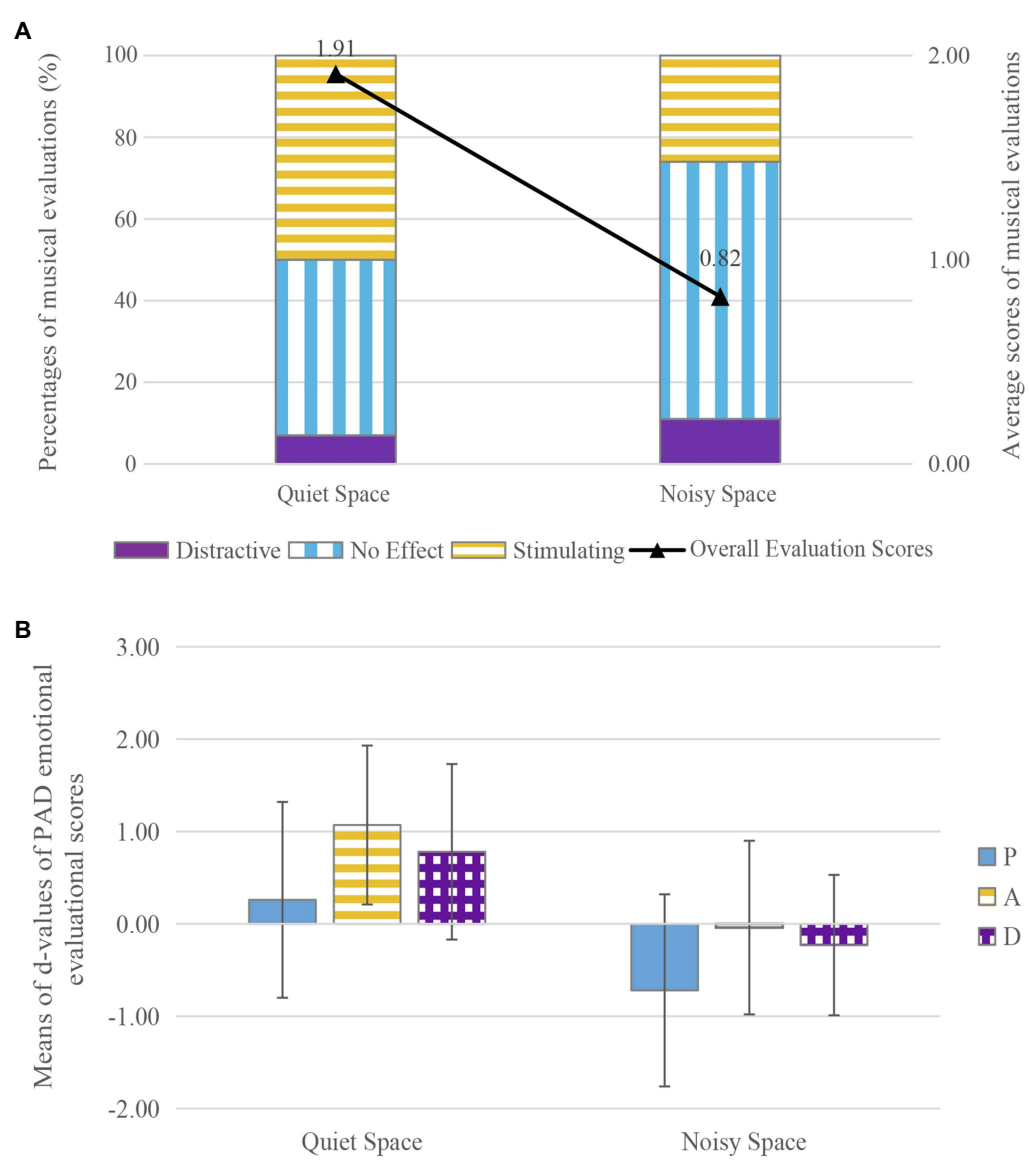
The effects of music on communication in spaces with different sound atmospheres: **(A)** the effects of music on musical evaluation, **(B)** the effects of music on PAD emotional evaluation.

In accordance with subjective evaluation of background sound under the uncontrolled condition, the experimental sites were sorted into quiet spaces and noisy spaces. FFR and CS (which had the same spatial function but a clear difference in sound atmosphere evaluation) were chosen for comparison. The relationships between sound atmosphere and percentages for musical evaluations (no effect/stimulating/distracting) and average scores for musical evaluations are shown in Figure 5A. The percentage of evaluations that rated the music as having a stimulating effect was much higher in the quiet space (50%) than in the noisy space (26%). The proportion of evaluations that rated the music as having no effect on communication was similar in two spaces (ranging from 7 to 11%). It was noticeable that a substantial proportion of communicators (63%) in noisy spaces thought that the music had no effect on communication. Average scores for musical evaluation were higher in the quiet space (*M*=1.91, *SD*=2.896) than in the noisy space (*M*=0.82, *SD*=3.402). The results of the *t*-test showed that there was no significant effect of sound atmosphere on musical evaluation (*t*=−1.801, *df*=109, *p*=0.074<0.1, *d*=0.34).

The average *d*-values for PAD emotional evaluations in spaces with different sound atmospheres are shown in Figure 5B. The *d*-values for pleasure, arousal, and dominance increased by 0.26 (*SD*=2.130), 1.07 (*SD*=1.725), and 0.78 (*SD*=1.910) in quiet spaces, but decreased by 0.72 (*SD*=2.085), 0.04 (*SD*=1.870), and 0.23 (*SD*=1.536) in noisy spaces. The results of the *t*-test showed that there was a significant effect of music on pleasure (*t*=2.446, *df*=109, *p*<0.05, *d*=0.46), arousal (*t*=3.243, *df*=109, *p*<0.01, *d*=0.62), and dominance (*t*=3.066, *df*=109, *p*<0.01, *d*=0.58) in spaces with different sound atmospheres. These findings indicate that music can be effective in enhancing pleasure, arousal, and dominance during communication in quiet spaces.

### Interior Furnishings

**Figure 6:**
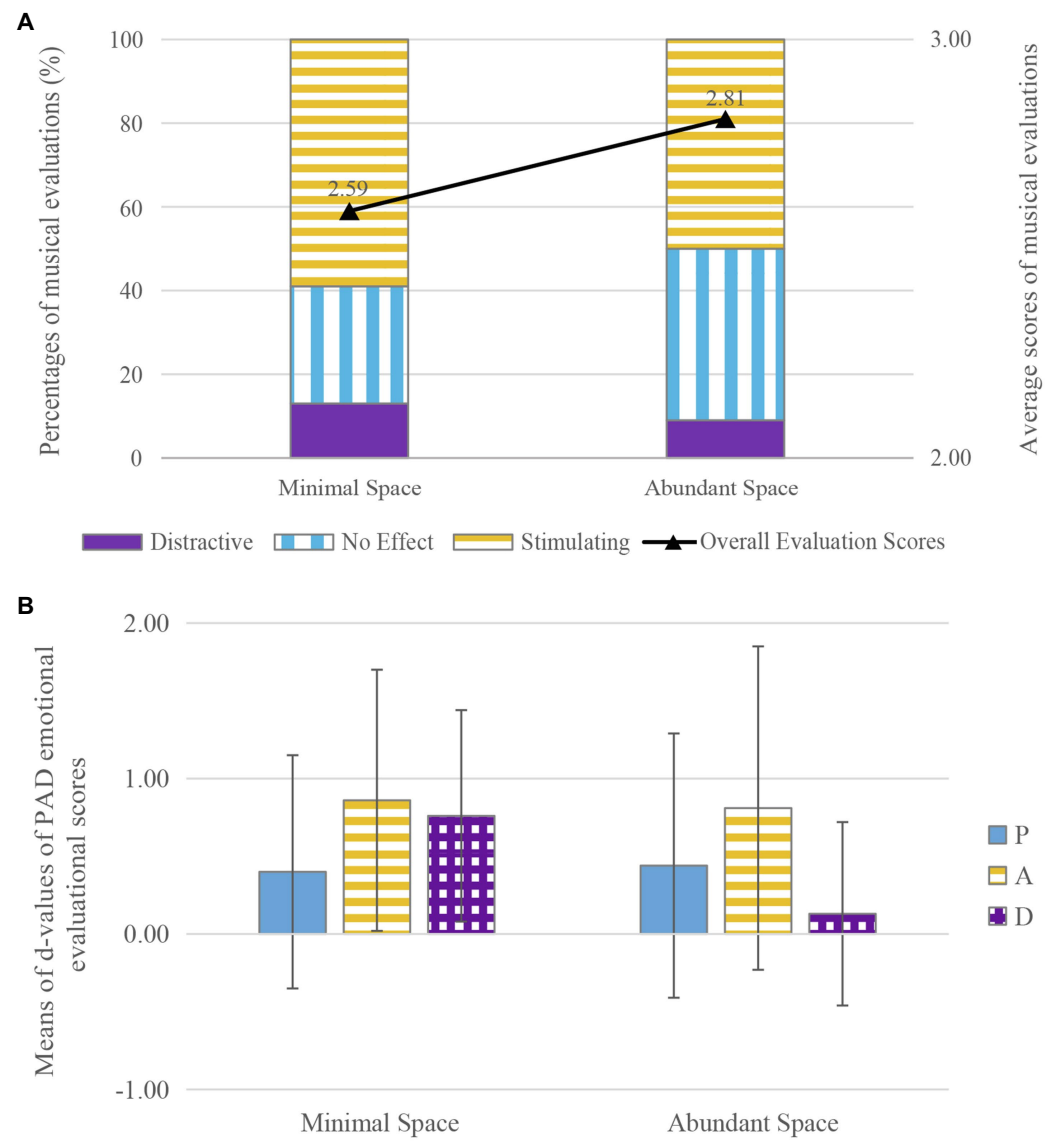
The effects of music on communication in spaces with different indoor furnishings: **(A)** the effects of music on musical evaluation, **(B)** the effects of music on PAD emotional evaluation.

Diversity of furniture is one of the main factors to evaluate the interior furnishings (Kaye and Murray, 1982). In this study, the experimental sites were sorted into minimal spaces (within three) and abundant spaces (more than three) in terms of the number of furniture types. DT and LR which had the same spatial function and visual and sound atmospheres but a clear difference in interior furnishings were chosen for comparison. The relationships between interior furnishings and the percentages for musical evaluations (no effect/stimulating/distracting) and the average scores for musical evaluations are shown in Figure 6A. The proportion of evaluations that rated the music as stimulating and as distracting were similar in the two groups (from 50 to 59% and from 9 to 13%, respectively). The percentage of evaluations that rated the music as having no effect was higher in the abundant space (41%) than in the minimal space (28%). Average scores for musical evaluation were slightly higher in the abundant space (*M*=2.81, *SD*=3.693) than in the minimal space (*M*=2.59, *SD*=3.853). The results of the *t*-test showed that there was no significant effect of interior furnishings on musical evaluation (*t*=−0.279, *df*=108, *p*=0.781, *d*=0.06).

The average *d*-values for PAD emotional evaluations in spaces with different interior furnishings are shown in Figure 6B. The *d*-values for pleasure and arousal were similar in the minimal space and abundant spaces, increasing by 0.40 (*SD*=1.515), 0.44 (*SD*=1.703), 0.86 (*SD*=1.673), and 0.81 (*SD*=2.086), respectively. The *d*-value for dominance was higher in the minimal space (*M*=0.76, *SD*=1.379) than in abundant spaces (*M*=0.13, *SD*=1.185). The results of the *t*-test showed that there was a significant effect of music on dominance in spaces with different interior furnishings (*t*=2.268, *df*=108, *p*<0.05, *d*=0.48). This finding indicates that the positive effect of music on dominance during communication was significant in the minimal space.

### Social Characteristics

Studies have found that social characteristics such as gender, age, and situation can lead to different emotion evaluations (Gabrielsson, 2002; Hunter et al., 2011; Schubert, 2013). In accordance with the behavioral mode of communication, this study analyzed the effects of music on participants with different intimacy levels, in different gender combinations and in groups of different sizes during communication. Based on the number of treatments, ANOVA was used to test for significant differences in the various intimacy levels (stranger, acquaintance, and close friend) of participants (Starzyk et al., 2006). Independent *t*-tests were performed to assess differences between different gender combinations and number of participants. The average scores of musical evaluation and the *d*-values of PAD emotional evaluation were summarized, and the SD was identified *via t*-tests. The results are shown in Table 4.

**Table 4:** Musical and emotional evaluations in terms of social characteristics.

					D-values for emotional evaluation		
Site	Statistical test	Social characteristic		Musical evaluation	Pleasure	Arousal	Dominance
Learning space	ANOVA	Intimacy level	Stranger	2.03	0.93	0.67	0.60
			Acquaintance	2.06	0.66	0.28	0.72
			Close friend	2.29	0.06	0.79	0.33
	*t*-test	Gender combination	Single gender	1.83 (SD = 3.679)	0.57 (SD = 1.839)	0.53 (SD = 2.052)	1.23 (SD = 2.179)
			Mixed gender	2.40 (SD = 3.231)	0.40 (SD = 2.052)	0.67 (SD = 2.155)	−0.02 (SD = 1.737)
		Group size	One-on-one	2.13 (SD = 3.553)	0.34 (SD = 1.873)	0.53 (SD = 2.276)	0.49 (SD = 2.062)
			Multi participants	2.26 (SD = 2.958)	0.96 (SD = 2.225)	0.91 (SD = 1.756)	0.61 (SD = 1.924)
Dining space	ANOVA	Intimacy level	Stranger	2.71	0.29	0.81	0.86
			Acquaintance	0.68	−1.04	−0.20	0.08
			Close friend	1.17	−0.11	0.68	0.14
	*t*-test	Gender combination	Single gender	1.02 (SD = 3.514)	−0.09 (SD = 2.003)	0.45 (SD = 1.768)	0.13 (SD = 1.585)
			Mixed gender	1.69 (SD = 2.834)	−0.40 (SD = 2.306)	0.56 (SD = 1.998)	0.40 (SD = 1.987)
		Group size	One-on-one	1.22 (SD = 3.037)	−0.62 (SD = 1.941)	0.12 (SD = 1.767)	0.02 (SD = 1.600)
			Multi participants	1.51 (SD = 3.402)	0.20 (SD = 2.324)	0.96 (SD = 1.918)	0.55 (SD = 1.973)
Residential space	*t*-test	Intimacy level	Acquaintance	3.97 (SD = 3.551)	0.97 (SD = 1.204)	1.00 (SD = 1.901)	1.22 (SD = 1.237)
			Close friend	1.31 (SD = 3.725)	0.18 (SD = 1.642)	0.78 (SD = 1.755)	0.37 (SD = 1.270)
		Gender combination	Single gender	2.08 (SD = 3.908)	0.44 (SD = 1.609)	0.92 (SD = 1.837)	0.60 (SD = 1.357)
			Mixed gender	2.11 (SD = 3.408)	0.11 (SD = 0.928)	0.00 (SD = 0.866)	0.78 (SD = 0.667)
		Group size	One-on-one	1.32 (SD = 3.913)	0.88 (SD = 1.509)	1.28 (SD = 1.768)	0.68 (SD = 1.435)
			Multi participants	2.31 (SD = 3.833)	0.27 (SD = 1.561)	0.72 (SD = 1.790)	0.60 (SD = 1.284)

The effects of music on communication varied in different functional indoor spaces when participants were with different social characteristics, such as intimacy level, gender combination, and group size of communicators.

In the learning space, there was a significant effect of music on dominance during communication in different gender combinations. The results of the *t*-tests showed that in comparison to mixed-gender groups (*M*=−0.02, *SD*=1.737), dominance (*t*=3.347, *df*=108, *p*<0.01, *d*=0.64) was enhanced by music in single-gender groups (*M*=1.23, *SD*=2.179).

In the dining space, compared to the intimacy levels of acquaintances and close friends, the average scores for musical evaluation were 1.54–2.03 higher in the group of strangers. The results of the ANOVA indicated that communication was promoted to some extent when participants were strangers (*df*1=2, *df*2=108, *F*=2.639, *p*=0.076<0.1, *d*=0.28), meanwhile, *d*-values of pleasure (*df*1=2, *df*2=108, *F*=2.541, *p*=0.083<0.1, *d*=0.48) and arousal (*df*1=2, *df*2=108, *F*=2.369, *p*=0.098<0.1, *d*=0.49) were higher in the stranger group. Further, all of the musical evaluation scores and *d*-values for emotional evaluations were higher when there were more than two participants, and the results of the *t*-tests show that there was an significant effect of music on pleasure (*t*=−2.008, *df*=109, *p*<0.05, *d*=0.38) and arousal (*t*=−2.412, *df*=109, *p*<0.05, *d*=0.46).

In the residential space, the average scores for musical evaluation were 2.66 higher in acquaintances compared to close friends. The results of the *t*-tests show that there was a significant effect of music on communication (*t*=3.448, *df*=108, *p*<0.01, *d*=0.72), and pleasure (*t*=−2.008, *df*=108, *p*<0.05, *d*=0.52) and dominance (*t*=−2.412, *df*=108, *p*<0.01, *d*=0.67) were efficiently enhanced by music in the group of acquaintances.

Therefore, in terms of social characteristics, there is evidence that when in the learning space, using music to promote communication is more suitable for single-gender groups. And when there are more than two communicators in the dining space, pleasure and arousal can be efficiently enhanced by music. In the residential space, when the intimacy level of roommates is not that close, music environment can be an effective way to promote communication.

## Discussion

This paper contributes to the body of research into the effects of audiovisual interaction on social interaction by comparing the effects of music on communication in different spaces in terms of function, visual atmosphere, sound atmosphere, and interior furnishings. In terms of spatial function, people have particular behavioral patterns in specific functional spaces (for instance, eating meals in a restaurant, or resting in a bedroom). In this study, spaces for learning, dining, and resting were chosen in accordance with the main activities of college students.

The results were inconsistent with Hypothesis 1; there were no significant differences between the effects of music on communication or emotion during communication in spaces with different functions. Post-experiment interview suggested that this result is to some extent attributable to the fact that chatting is a common behavior pattern in all the spaces, regardless of their primary function, and thus all the sites were suitable for communication. Compared to spatial function, the degree of privacy offered by a space may have a more significant effect on communication. Numerous studies have explored the need for privacy and intelligibility of speech in different spaces, such as open-plan offices, hospitals, and residences (Cavanaugh and Hirtle, 2006; Virjonen et al., 2007; Roy, 2017), finding that people’s satisfaction is closely related to speech privacy. To date, most studies have focused on the analysis of emotional feelings in specific spaces, with little horizontal comparison across different functional spaces. Thus, the results of this part of the present study provide a point of reference for future research.

For Hypothesis 2 on visual atmosphere, the results show that music had greater effects on communication in the warm-toned spaces than in the cold-toned spaces. However, there were no significant differences in the effects of music on pleasure, arousal, or dominance in spaces with different visual atmospheres. The effect size of the PAD emotional evaluations did not seem to match the overall effects of music on communication. Previous studies indicate that experiences of color have their roots in conscious, subconscious, and unconscious processes of human behaviors (Reddy et al., 2012) and that the effect of music on emotional intervention plays a dominant role, followed by color (Li et al., 2018). The present results may be due to the fact that music has a greater effect on emotion than color does. Against a given musical background, it may be difficult for participants to distinguish differences in the emotion during communication in spaces with different visual atmospheres, and it is likely that the complex influence mechanism of color on emotion cannot be expressed simply in terms of a three-dimensional emotion model.

In terms of sound atmosphere, the subject of Hypothesis 3, the results indicate that communication can be enhanced by music in the quiet space to some degree, although, most of the communicators thought that there was almost no effect of music on communication in the noisy space. In the presence of music during communication, pleasure, arousal, and dominance all increased in the quiet space but decreased in the noisy space. These results are in line with previous research, which found that music plus ambient noise at comfortable levels of volume increases dining pleasure, while no music or a sound environment with music that is too loud has negative effects (Novak et al., 2010). It is worth noting that noise has both negative and positive aspects, and the absence of negative sound does not necessarily create a positive environment (Iyendo, 2016). Torresin et al. (2019) conducted a systematic review of positive indoor soundscapes; among their findings, specific sound types (i.e., natural sounds and sounds from residential areas) were found to reduce annoyance caused by disturbing tonal noises. Likewise, research exploring the acoustic environment of nursing homes showed that an environment that is rich and varied in sound sources tends to perform better in terms of safety and intimacy, as well as appropriateness, compared to monotonous and uneventful soundscapes (Aletta et al., 2017). Furthermore, the results of previous research have shown that musical evaluation during communication decreases sharply (from 1.31 to −2.13) when SPL exceeds 50dBA, becoming negative when SPL reaches 60dBA (Meng et al., 2020). Therefore, neither playing music at an appropriate volume in a noisy space nor playing it at over 60dBA in a quiet space is likely to improve the quality of communication.

For the evaluations in regarding minimal and abundant interior furnishings, the subject of Hypothesis 4, the results indicate that there were no significant differences between the effects of music on communication in spaces that had been furnished differently, but that music can enhance dominance during communication in the minimally furnished space. This finding may be due to the fact that complicated furnishings can distract people’s attention. A related study of the effects of commercial spatial factors on shopping behavior drew a similar conclusion; a complex grocery store environment was associated with low levels of pleasantness, delays in purchasing or even departures, as high levels of complexity can produce avoidance behaviors (Gilboa and Rafaeli, 2003). Differences in the arrangement as well as the density of furniture have been studied, showing that manipulation of furniture density may nullify the effects of arrangement; that is, with increasing density there is a corresponding decrease in distance between chairs, which is associated, up to a point, with increasing intimacy and friendliness (Kaye and Murray, 1982).

Communicators in the same seats had different spatial perceptions of scale that bore no clear relation to the indoor area. Take, for example, the learning corridor, which 53% of participants thought of as a large-scale space, whereas 47% held the opposite view. According to post-experiment interviews, the ratio of the length and width of a space, the density of crowds and furniture, and the enclosure mode of a space all affect the perception of spatial scale.

For Hypothesis 5, which focused on social characteristics, the results indicate that music had a different effect on communication when participants diverged in their intimacy levels, gender combinations and group size in different indoor spaces. In terms of intimacy level, the results showed the potential of music to strengthen social bonds in residential spaces. Compared to close friends, there was significant effect of music on acquaintances during communication, pleasure and dominance were effectively enhanced by music. Consistent with Hypothesis 5, Bronwyn et al. (2014) revealed the mechanism by which music enhances social bonds, indicating that listening to music facilitates endorphin release, which plays a central role in the maintenance of non-sexual, non-kinship social bonds. In this vein, roommate conflict is a key social problem in college (Sillars, 1980), and effective communication can enhance relationships among roommates (Wang et al., 2012). Music can thus be seen as an effective way to ease dormitory conflict when roommates have a low intimacy level.

In terms of gender combination, the results indicate that dominance during communication was significantly enhanced by music for single-gender groups in the learning spaces. Numerous studies have explored the effects of gender composition on social interactions such as cooperation, group discussion, and group learning among children (Smith-Lovin, 1989; Ausch, 1994; Willoughby et al., 2009). For example, with respect to group learning, more collaborative behaviors have been found in mixed-gender than in single-gender groups (Willoughby et al., 2009). Considering these findings in combination of the *d*-values of dominance in the learning spaces shown in Table 4, music can be considered an effective strategy to enhance dominance within single-gender groups, and to reduce differences in groups with gender combinations, during communication.

In terms of group size, the results show that music had greater effects on pleasure and arousal during communication when there were more than two participants in the dining spaces. A study of the effects of dining style on communication in restaurants indicated that when there were four or more diners per table, conversation increased compared to when there were fewer people, and frequency of conversation in centralized style (diners sharing a dish, such as a hot pot) was higher than in the separate (diners do not share dishes with others but eat their own food) with background music (Meng et al., 2017). Therefore, further confirmation of the most appropriate group size for communication in dining spaces is needed; in addition, dining style should be considered.

## Summary and Conclusion

Under the comprehensive pressures of academic and daily life, social anxiety is a widespread problem among college students, a particularly vulnerable group. Reliance on the Internet is an important factor in mental health, and the identification of ways to ease social barriers and to promote face-to-face communication is an important area of research. Using objective measurements of the natural sound environment and a subjective questionnaire survey of musical evaluations and PAD emotional evaluations during communication, this study examined the influence of music on emotion during communication in different indoor spaces and reached a number of conclusions. First, there were no significant differences in musical or emotional evaluation during communication in different functional spaces. Second, the positive effects of music were higher in warm-toned spaces, whereas the differences in the effects of music on emotion during communication in spaces with different visual atmospheres were not significant. Third, music had a significant effect on both musical evaluation and emotion during communication in spaces with different sound atmospheres, indicating that it can promote communication in quiet spaces. Fourth, in terms of interior furnishings, music in simply furnished spaces can enhance dominance. The effects of music on dominance were also higher in single-gender groups than in mixed-gender groups.

## Limitations

The present study can help improve communication by regulating spatial factors with music in terms of visual atmosphere, sound atmosphere, and interior furnishings. However, the study has a number of limitations which indicate that certain questions need to be discussed further.

There are rich varieties of musical emotions, and musical emotion characteristics can be classified based on musical dimensions such as pitch height, loudness, timbre, tempo, and intensity (Wieczorkowska et al., 2006). For example, Hevner (1936) presented eight synonym clusters to describe emotional perceptions of music, including happiness, gracefulness, sereneness, dreaminess, sadness, dignity, vigorousness, and excitement. The level of emotional perception of music varies with regard to different musical emotions (Baumgartner et al., 2006), and the emotion evoked by music is not always consistent with the perceived emotion (Gabrielsson, 2002). However, only one excerpt of peaceful music was used in the given experimental settings, and future research should seek to combine a wider range of music with different types of spaces.

In terms of the selection of experimental sites, limitations in terms of being able to control variables when comparing two spaces might have caused the results to be confounded by uncontrolled factors. In addition to the variables listed in this research, the spatial scale, dynamism, and indoor partitions are key factors of spatial perceptions and thus impact emotions (Hogg et al., 2011; Maheshwari et al., 2019). For instance, small rooms are considered more pleasant, calmer, and safer than large rooms (Tajadura-Jiménez et al., 2010). Regarding the visual atmosphere, the colors of the spaces were simply divided into warm-toned and cold-toned, while color harmony, as well as saturation and brightness, of color also impact emotion (Shen et al., 2015). In order to minimize the effects of uncontrolled spatial factors, virtual reality (VR) technology that provides a realistic and immersive environment (Chamilothori et al., 2019) can be considered for use in further studies.

Emotions are internal and mostly conscious, and self-report is an accepted way to evaluate them (Bradley and Lang, 1994). However, individuals have no direct access to the causal connections between external forces and internal responses – they are simply limited in their ability to track the complex causal story of their emotions (Russell, 2003). In addition, self-reports frequently refer to a certain period experienced in the past, and usually only salient single moments of the episode overall are emphasized (Fredrickson and Kahneman, 1993; Fiebig et al., 2020); thus, retrospective biases also need to be taken into account (Robinson and Clore, 2002). As the results of this study are based on self-reports, future studies should collect data regarding physiological changes to support the above findings.

## Data Availability Statement

The raw data supporting the conclusions of this article will be made available from the corresponding author upon reasonable request.

## Ethics Statement

The studies involving human participants were reviewed and approved by ethics committee, School of Architecture, Harbin Institute of Technology. Written informed consent for participation was not required for this study in accordance with the national legislation and the institutional requirements.

## Author Contributions

JJiang participated in the investigation, data curation, and writing of the original draft. QM provided conceptualization and participated in writing and editing the manuscript and in funding acquisition. JJi participated in the methodology, validation, and formal analysis. All authors contributed to the article and approved the submitted version.

## Funding

This research was funded by the National Natural Science Foundation of China (NSFC), grant numbers 51878210, 51678180, and 51608147, the Natural Science Foundation of Heilongjiang Province, grant number YQ2019E022 and the Open Projects Fund of Key Laboratory of Ecology and Energy-saving Study of Dense Habitat (Tongji University), Ministry of Education (2020030103).

## Conflict of Interest

The authors declare that the research was conducted in the absence of any commercial or financial relationships that could be construed as a potential conflict of interest.

## Publisher’s Note

All claims expressed in this article are solely those of the authors and do not necessarily represent those of their affiliated organizations, or those of the publisher, the editors and the reviewers. Any product that may be evaluated in this article, or claim that may be made by its manufacturer, is not guaranteed or endorsed by the publisher.
